# OP71 Treatment Patterns, Health Resource Utilization, And Costs Of Patients With Diffuse Large B-Cell Lymphoma: A Real-World Analysis

**DOI:** 10.1017/S026646232510127X

**Published:** 2025-12-29

**Authors:** Jorge Daza, Virginia Abello, Natalia Sánchez, Jorge Ceballos, Paola Omaña, Carlos Gómez, Daniel Samacá, Andrés F. Cardona, Sergio Beltran

## Abstract

**Introduction:**

Diffuse large B-cell lymphoma (DLBCL) is the most common hematological neoplasm in adults globally and in Colombia, but there are limited data on its local economic impact. This study analyzed the relationship between clinical variables and healthcare resource utilization of patients with DLBCL in a Colombian health maintenance organization.

**Methods:**

This analytical observational study included patients with histopathologically confirmed DLBCL treated at the Luis Carlos Sarmiento Angulo Cancer Treatment and Research Center (CTIC) between July 2022 and September 2025. We reported an interim analysis with a data cut-off in July 2024. Clinical data from CTIC’s institutional registry were merged with the information provided by the administrative database, covering healthcare resource use and costs for drugs, procedures, and medical supplies. A multivariate linear regression estimated the effect of prognosis (revised International Prognostic Index [R-IPI] score) on total costs, adjusting for confounding (e.g., sex, comorbidities, and history of neoplasm).

**Results:**

Sixty-two patients were included (mean 66 years [standard deviation 14]; 58% men). R-IPI scores at baseline indicated that 34 percent were at high-intermediate risk, 21 percent had low and low-intermediate risk, and 23 percent were at high risk. R-IPI scores at baseline showed that 17.7 percent patients had very good prognosis, while 24.2 and 41.9 percent had good or poor prognosis, respectively (16.1% missing). The R-CHOP chemotherapy regimen was the most common first-line (1L) treatment (73%), and fifteen percent of patients had disease progression. The total 1L treatment cost was COP1,386,178,104 (USD320,500) per patient. Thirteen patients received second-line (2L) treatment, costing COP3,203,033,513 (USD740,577) per patient. Regression analysis showed that patients with poor prognosis, per R-IPI score, incurred 8.9 times higher costs than those with very good R-IPI scores.

**Conclusions:**

Our study showed the association between clinical variables and the cost of treatment in patients with DLBCL. Regression analysis indicated that higher R-IPI scores and advanced disease stages significantly increased costs. The cost per patient for 2L treatment was 2.3 times higher than for 1L treatment, highlighting the importance of timely detection and treatment.

